# Draft Genome Sequence of Antarctic Psychrotroph Streptomyces fildesensis Strain INACH3013, Isolated from King George Island Soil

**DOI:** 10.1128/MRA.01453-20

**Published:** 2021-02-04

**Authors:** Paris Lavin, Carlos Henríquez-Castillo, Sheau Ting Yong, Daniel Valenzuela-Heredia, Romulo Oses, Katherine Frez, Marcela P. Borba, Cristina Purcarea, C. M. V. L. Wong

**Affiliations:** a Departamento de Biotecnología, Facultad de Ciencias del Mar y Recursos Biológicos, Universidad de Antofagasta, Laboratorio de Complejidad Microbiana y Ecología Funcional, Instituto Antofagasta, Universidad de Antofagasta, Antofagasta, Chile; b Laboratorio de Fisiología y Genética Marina (FIGEMA), Centro de Estudios Avanzados de Zonas Áridas (CEAZA), Coquimbo, Chile; c Facultad de Ciencias del Mar, Universidad Catolica del Norte, Coquimbo, Chile; d Biotechnology Research Institute, Universiti Malaysia Sabah, Kota Kinabalu, Sabah, Malaysia; e Facultad de Ingeniería y Ciencias, Universidad Adolfo Ibáñez, Viña del Mar, Chile; f Centro Regional de Investigación y Desarrollo Sustentable de Atacama (CRIDESAT), Universidad de Atacama, Copiapó, Chile; g Laboratório de Microbiologia Aplicada, Instituto de Ciências Básicas da Saúde, Departamento de Microbiologia, Imunologia e Parasitologia, Porto Alegre, Brazil; h Department of Microbiology, Institute of Biology, Bucharest, Romania; Georgia Institute of Technology

## Abstract

The draft genome sequence of Streptomyces fildesensis strain INACH3013, a psychrotrophic bacterium isolated from Northwest Antarctic soil, was reported. The genome sequence totaling 9,306,785 bp resulted from 122 contigs characterized by a GC content of 70.55%.

## ANNOUNCEMENT

*Streptomyces* species are aerobic, spore-forming, filamentous Gram-positive bacteria with large and linear chromosomes characterized by a high GC content (1). Members of this genus are ubiquitous in a broad range of habitats, including extreme environments ranging from tropical to polar regions (2), and are known for their ability to synthesize a wide variety of biologically active secondary metabolites (3).

Here, we report the partial genome sequence of Streptomyces fildesensis strain INACH3013, isolated from soil of the Antarctic Fildes Peninsula (62°12′26.4″S, 58°58′28.7″W) (2, 4). The taxonomic status of the isolate was established in reference 2. This strain shows antibacterial compound production at temperatures below the optimal growth value (28°C) and a temperature-dependent trade-off between secondary compound production and growth (4).

*S. fildesensis* strain INACH3013 was cultured in triplicate using 500 ml of oatmeal liquid medium (20 g liter^−1^) with agitation (180 rpm) for 5 days at 12°C ± 1°C. Genomic DNA was isolated from a single colony using a DNeasy blood and tissue kit (Qiagen, Inc., USA), according to the manufacturer’s procedure. The taxonomic affiliation of the strain was confirmed by partial sequencing of the 16S rRNA gene (NCBI accession number KJ624755). The total DNA was processed by using a TruSeq DNA PCR-free library preparation kit and HiSeq 2000 paired-end sequencing (Illumina, Inc., San Diego, CA). The corresponding genome sequencing generated 9,445,886 short reads with an average length of 100 nucleotide bases. The sequence reads were filtered using Trimmomatic v0.39 (5) using default parameters. The *de novo* genome assembly was performed using SPAdes 3.14.1 with default parameters (6). The partial genome sequence was assembled from 73 large contigs (>10,000 bp) and 49 small contigs (<10,000 bp), with an *N*_50_ value of 152,215 bp, with the largest contig of 681,566 bp. The *S. fildesensis* strain INACH3013 genome sequence had an estimated completion of 99.28% (7) and a mean coverage of 32.7025×. The draft genome was annotated with the NCBI Prokaryotic Genome Annotation Pipeline (PGAP) using default settings (8). The final assembly had a GC content of 70.55%, with 8,622 protein-coding genes, 4 rRNAs, 77 tRNAs, and an average gene length of 957.88 bp. antiSMASH v5.0 (9) predicted a total of 32 biosynthetic gene clusters.

### Data availability.

This whole-genome shotgun project has been deposited at DDBJ/ENA/GenBank under the accession number JAAIKO000000000. The version described in this paper is the first version, JAAIKO010000000. The SRA accession number is SRX7554417.
